# Case Report: Detection of Symptomatic Treatment-Related Changes in a Patient With Anaplastic Oligodendroglioma Using FET PET

**DOI:** 10.3389/fonc.2021.735388

**Published:** 2021-11-16

**Authors:** Elena Katharina Bauer, Jan-Michael Werner, Gereon R. Fink, Karl-Josef Langen, Norbert Galldiks

**Affiliations:** ^1^ Department of Neurology, Faculty of Medicine and University Hospital Cologne, University of Cologne, Cologne, Germany; ^2^ Institute of Neuroscience and Medicine (INM-3, -4), Research Center Juelich, Juelich, Germany; ^3^ Department of Nuclear Medicine, University Hospital Aachen, Aachen, Germany; ^4^ Center of Integrated Oncology (CIO), Universities of Aachen, Bonn, Cologne, and Duesseldorf, Cologne, Germany

**Keywords:** amino acid PET, pseudoprogression, chemoradiation, oligodendroglioma, diagnostic challenge

## Abstract

Following local and systemic treatment of gliomas, the differentiation between glioma relapse and treatment-related changes such as pseudoprogression or radiation necrosis using conventional MRI is limited. To overcome this limitation, various amino acid PET tracers such as *O*-[2-(^18^F)-fluoroethyl]-L-tyrosine (FET) are increasingly used and provide valuable additional clinical information. We here report neuroimaging findings in a clincally symptomatic 53-year-old woman with a recurrent anaplastic oligodendroglioma with MRI findings highly suspicious for tumor progression. In contrast, FET PET imaging suggested treatment-related changes considerably earlier than the regression of contrast enhancement on MRI. In patients with oligodendroglioma, the phenomenon of symptomatic treatment-related changes is not well described, making these imaging findings unique and important for clinical decision-making.

## Introduction

Treatment-related changes on conventional MRI are predominantly characterized by both an increase of contrast enhancement in the T1-weighted image and a hyperintensity in the fluid-attenuated inversion recovery (FLAIR) sequence, which either remain stable or even disappear over time without any change of anticancer treatment. Not infrequently, patients experience no clinical deterioration. Misinterpretation of these imaging findings may result in an unnecessary discontinuation of an effective treatment with a potentially negative impact on the patient’s survival. Due to the low specificity of conventional MRI, the differentiation of treatment-related changes from actual tumor progression in glioma patients following radiotherapy or chemoradiation using conventional MRI is challenging (1–4).

The additional use of amino acid PET using O-[2-(^18^F)-fluoroethyl]-L-tyrosine (FET) in the care of glioma patients is gaining more attention. In recent years, a high diagnostic accuracy for the differentiation between tumor progression and treatment-related changes using FET PET has repeatedly been shown predominantly in patients with astrocytic gliomas (1, 2, 5, 6). In these studies, either static parameters or the combination of static and dynamic parameters derived from FET PET seem to be of considerable clinical value for the for the differentiation of tumor progression from treatment-related changes (range of sensitivity, 91-99%; range of specificity, 94-100%) (1, 5, 6). Despite its clinical relevance, only a few studies described treatment-related changes in patients with oligodendrogliomas.

Oligodendroglial gliomas are characterized by the presence of an isocitrate dehydrogenase (IDH) gene mutation and a 1p/19q codeletion and are histologically corresponding to the grade II or III of the in 2016 revised World Health Organisation (WHO) classification of Tumors of the Central Nervous System (7). This rare tumor entity accounts for 5.3% of all tumors of the central nervous system.

We here report the phenomenon of symptomatic treatment-related changes in a patient with an anaplastic oligodendroglioma.

## Case Presentation

**Figure 1:**
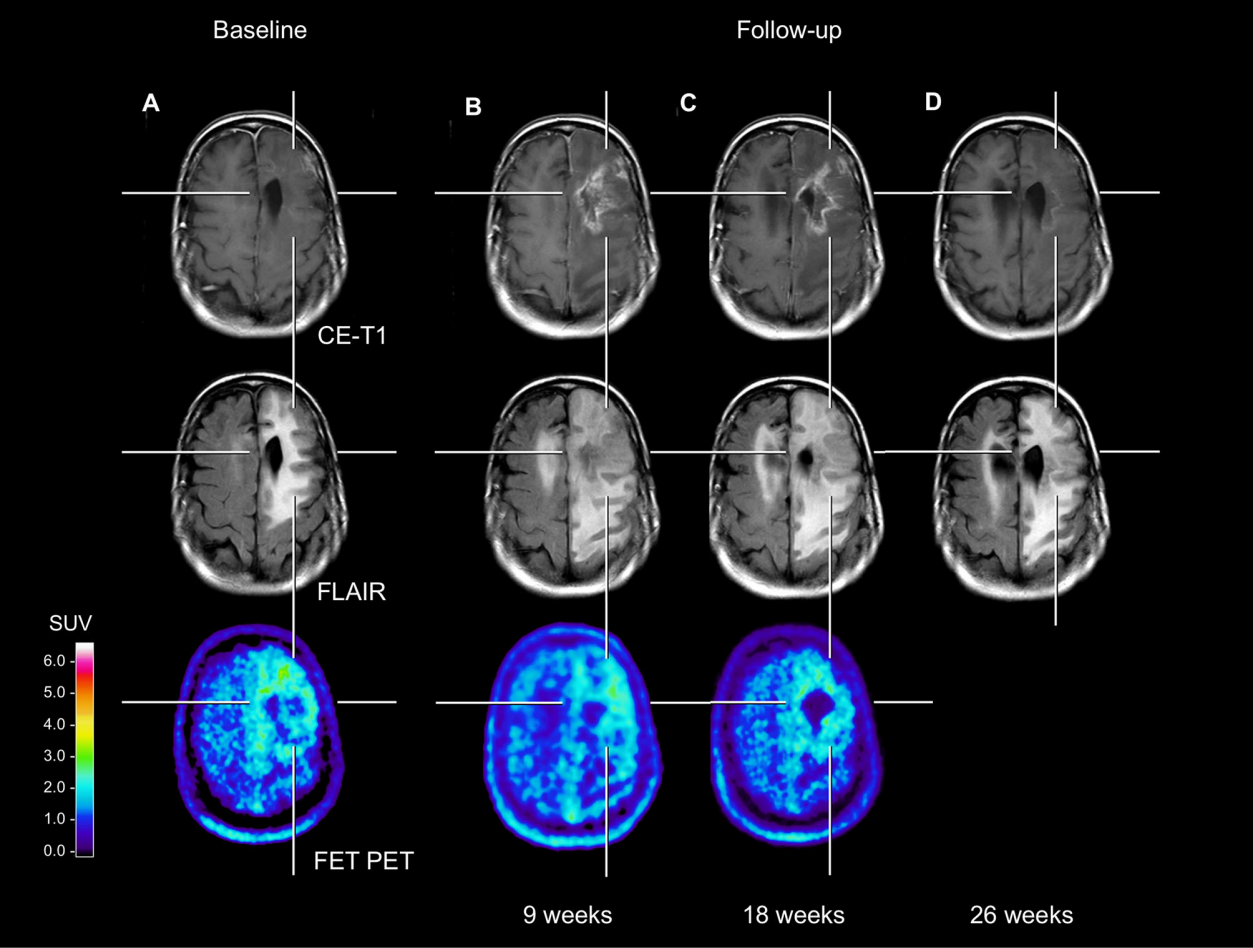
Neuroimages including contrast-enhanced MRI, FLAIR-weighted MRI, and FET PET of a patient with an anaplastic oligodendroglioma (IDH mutant, 1p/19q co-deleted). Compared to the baseline MRI **(A)**, the follow-up MRI perfomed 9 weeks later (17 weeks after end of radiotherapy) **(B)** showed a substantial contrast enhancement and an extensive FLAIR signal hyperintensity in the area of the resection defect. In contrast, both the baseline and first follow-up FET PET **(A, B)** showed no pathologically increased tracer uptake (mean tumor-to-brain ratios in both scans, 1.8). The rationale for this is based on the fact that several FET PET studies suggested that especially in pretreated glioma patients a mean tumor-to-brain ratio of 2.0 separates best between tumor relapse and treatment-related changes (1, 6, 8). The second follow-up MRI **(C)** showed a further slight increase of the FLAIR signal alteration. Again, the corresponding follow-up FET PET showed no substantial change of metabolic activity compared to the previous examinations **(A, B)**. Not earlier than 26 weeks, the third follow-up MRI **(D)** revealed an almost complete decrease of the contrast enhancement.

A 53-year-old female patient with a recurrent anaplastic oligodendroglioma (IDH mutant, 1p/19q co-deleted) was diagnosed with the second local tumor recurrence 15 years after initial diagnosis. The first-line treatment included tumor resection and radiotherapy. Eleven years later, a first local tumor recurrence was treated with re-resection followed by adjuvant temozolomide chemotherapy. Four years later, the tumor recurred locally again. Following complete resection and re-irradiation, she underwent adjuvant nitrosourea-based chemotherapy with procarbazine and lomustine. After two cycles, the patient experienced a clinical deterioration with an expressive aphasia and a right-sided hemiparesis. According to current response assessment criteria (3), the findings of the first and second follow-up MRI were consistent with “Progressive Disease”, whereas the corresponding FET PET scans remained unchanged compared to baseline (see **Figure 1**). Without changing the treatment regimen, symptomatic therapy with dexamethasone prompted a rapid clinical improvement. During follow-up, dexamethasone could subsequently be tapered from 8 mg to 0.5 mg. The diagnosis of treatment-related changes was based on the MRI findings performed 26 weeks after baseline imaging, which showed especially an almost complete decrease of contrast enhancement, accompanied by a further improvement of the clinical condition.

## Discussion

Following radiotherapy with or without alyklating chemotherapy, treatment-related changes such as pseudoprogression or radiation necrosis represent a major diagnostic challenge in patients with astrocytic glioma. In this group of brain tumors, the steadily increasing number of studies highlights the value of amino acid PET for the differentiation of treatment-related changes from actual tumor progression with a high diagnostic accuracy (1, 2, 4, 9).

On the other hand, oligodendrogliomas represent a small glioma subgroup and constitute only approximately 5% of primary intracranial tumors. Importantly, it has been demonstrated that in oligodendrogliomas the rate of treatment-related changes is significantly lower than in patients with gliomas without 1p/19q codeletion (i.e., astrocytoma, glioblastoma) (10, 11). For example, Lin and co-workers reported a pseudo-progression rate of 31% for tumors without codeletions, and only 3% for tumors with codeletions (11). Moreover, amino acid uptake in 1p/19q-codeleted oligodendroglial tumors is generally significantly increased, even higher when compared to IDH mutant astrocytic tumors (12, 13), and similar to glioblastoma (13). Thus, a considerably increased FET uptake can be expected in the case of oligodendroglioma relapse. Consequently, the absence of this uptake pattern in our case further supports the diagnosis of treatment-related changes.

Another important aspect of the present case is the extensive increase of the FLAIR signal hyperintensity, suggesting non-enhancing tumor progression. In contrast, FET PET showed in spatial correspondence no pathological increased tracer uptake. Thus, FET PET seems also to be of value to rule out this particular phenomenon. In summary, this case highlights the value of FET PET for the diagnosis of treatment-related changes in a glioma subtype characterized by a naturally increased amino acid uptake and a low rate of treatment-related changes.

## Data Availability Statement

The original contributions presented in the study are included in the article/supplementary material. Further inquiries can be directed to the corresponding author.

## Ethics Statement

All procedures performed in the studies involving human participants were in accordance with the ethical standards of the institutional and/or national research committee and with the 1964 Helsinki Declaration and its later amendments or comparable ethical standards. The patients/participants provided their written informed consent to participate in this study. Written informed consent was obtained from the individual(s) for the publication of any potentially identifiable images or data included in this article.

## Author Contributions

Data acquisition, EB, NG, and J-MW. Writing of manuscript drafts, EB, NG, and J-MW. All authors contributed to the article and approved the submitted version.

## Funding

Supported by the Deutsche Forschungsgemeinschaft (DFG), project number 428090865 (EB and NG), and the Cologne Clinician Scientist Program (CCSP)/Faculty of Medicine/University of Cologne, funded by the DFG, FI 773/15-1 (J-MW).

## Conflict of Interest

The authors declare that the research was conducted in the absence of any commercial or financial relationships that could be construed as a potential conflict of interest.

## Publisher’s Note

All claims expressed in this article are solely those of the authors and do not necessarily represent those of their affiliated organizations, or those of the publisher, the editors and the reviewers. Any product that may be evaluated in this article, or claim that may be made by its manufacturer, is not guaranteed or endorsed by the publisher.
